# High-Throughput Sequencing Reveals Composition, Diversity, and Functional Prediction of Root-Associated Microbial Communities of Dominant Plants in an Ecologically Sensitive Area of the Loess Plateau

**DOI:** 10.3390/microorganisms14092002

**Published:** 2026-09-09

**Authors:** Gexue Bai, Qingqing Tan, Bingbing Han, Ruidong Li, Lijun Gu, Xiaojing Wang, Jie Zhang, Yan Li, Quanfang Zhang

**Affiliations:** 1 The Second Geological and Mineral Exploration Institute of Gansu Provincial Bureau of Geology and Mineral Exploration and Development, Lanzhou 730020, China; baigexue6009@163.com; 2 Shandong Academy of Agricultural Sciences, Jinan 250100, China; tanqingqing88@163.com; 3 Gansu Institute of Engineering Geology, Lanzhou 730000, China; 13519653867@163.com (B.H.); 13919456599@163.com (R.L.); 4 College of Pastoral Agriculture Science and Technology, Lanzhou University, Lanzhou 730020, China; gulj@lzu.edu.cn; 5 Institute of Crop Research, Tianjin Academy of Agricultural Sciences, Tianjin 300381, China; 210-wxj@163.com; 6 State Key Laboratory of Green Papermaking and Resource Recycling, Qilu University of Technology (Shandong Academy of Sciences), Jinan 250353, China; zhangjie@qlu.edu.cn

**Keywords:** alpine mining area, ecological restoration, rhizosphere microorganisms, high-throughput sequencing, community diversity, functional microbial groups

## Abstract

The alpine mining area of the Qilian Mountains features a fragile ecosystem, severe soil degradation due to mining disturbances, and slow natural recovery. To clarify the ecological restoration potential of rhizosphere microorganisms associated with native dominant plants, this study investigated the community structure, diversity, and functional differentiation patterns of rhizosphere bacterial and fungal communities associated with seven native dominant plant species in the Tianzhu mining area. Using Illumina NovaSeq 6000 high-throughput sequencing, we amplified the bacterial 16S rRNA V3–V4 region and the fungal ITS1 region. A total of 24 sequencing libraries (seven plant species + bare soil, three replicates each) were analyzed. Bioinformatics analyses examined ASV distributions, alpha/beta diversity, species composition, and differentially enriched taxa, followed by functional predictions. Bacterial alpha diversity (Chao1: 623.39–1553.25; Shannon: 7.49–9.67) varied significantly among plant species, with *Allium przewalskianum* (AP) showing the highest bacterial richness and diversity (Chao1 = 1553.25 ± 35.89, Shannon = 9.67 ± 0.02). Fungal alpha diversity also showed significant variation (Chao1: 69.66–488.56; Shannon: 3.24–4.96), with *Dasiphora fruticosa* (DF) exhibiting the highest fungal diversity (Chao1 = 488.56 ± 18.71, Shannon = 7.32 ± 0.06). At the phylum level, *Proteobacteria* (32.22–50.32%) and *Actinobacteriota* (15.04–23.19%) were core bacterial groups, and *Ascomycota* (45.54–96.07%) dominated fungal communities. PERMANOVA confirmed significant differences in community composition among plant species (bacteria: R^2^ = 0.78, *p* < 0.001; fungi: R^2^ = 0.84, *p* < 0.001). Different plant species were associated with distinct predicted functional taxa, which may serve as candidate biomarkers for soil remediation. However, all functional interpretations are predictive and require experimental validation. In conclusion, rhizosphere microbial community composition and predicted functional profiles differed among native plant species, providing correlative evidence and candidate targets for future vegetation–microbe synergy studies in alpine mining area restoration.

## 1. Introduction

Mineral resource development serves as an important pillar for regional socio-economic development. However, high-intensity and large-scale mining activities can readily disrupt regional ecological balance, triggering a series of eco-environmental problems including soil structural fragmentation, nutrient depletion, acid–base imbalance, heavy metal enrichment, and vegetation degradation. These issues substantially reduce the stability and self-recovery capacity of mining area ecosystems, posing a serious threat to regional ecological security [1]. Tianzhu Tibetan Autonomous County is located at the eastern end of the Qilian Mountains, where the convergence of three major plateaus creates a complex continental plateau climate, rendering the regional ecological foundation sensitive and fragile, and making it difficult for damaged ecosystems to achieve natural recovery in the short term [2]. Long-term mining activities have formed typical degraded habitats, and the synergistic effects of soil impoverishment, pollutant accumulation, and nutrient imbalances further impede the ecological restoration process [3,4]. In such extremely degraded habitats, natural recovery proceeds slowly, making efficient and sustainable artificially assisted ecological restoration a core requirement for ecological management in alpine mining areas [5,6].

Vegetation reconstruction and soil restructuring are core components of ecological restoration in mining areas. Compared with exotic species, native pioneer plants, owing to their strong stress tolerance, have become preferred materials for vegetation restoration [7,8]. Through long-term natural succession, the Tianzhu mining area has fostered a number of stably growing native dominant plant populations. These plants not only consolidate soil and conserve water and prevent soil erosion, but their root-mediated rhizosphere microecosystems also serve as the core driving force for ameliorating degraded soils, activating soil biological activity, and promoting positive ecosystem succession [9,10]. Plants maintain close symbiotic interactions with rhizosphere microorganisms, which can assist plant adaptation to mining stress by mobilizing nutrients, secreting growth-promoting metabolites, degrading pollutants, and enhancing stress resistance. Rhizosphere microorganisms are thus key biotic factors sustaining vegetation colonization and community stability [11].

The unique alpine climate, geological conditions, and degraded soil have shaped distinctive rhizosphere microbial community structures of plants in the Tianzhu mining area. However, the synergistic adaptation mechanisms between dominant plants and their rhizosphere microorganisms, as well as microbial functional differentiation patterns, remain unclear. A systematic analysis of the assembly characteristics and functional differences in rhizosphere microbial communities associated with native dominant plants can advance the theoretical framework of plant–microbe interactions in alpine mining areas and provide core microbial strain resources and data support for the development of bioremediation technologies in mining regions. High-throughput sequencing technology, owing to its advantages in efficiently and accurately resolving microbial community structures, has been widely applied in rhizosphere microecology research, offering reliable technical support for systematically revealing the microbial diversity and functional characteristics of degraded soils in mining areas [12,13].

This study aims to elucidate the rhizosphere microecological characteristics of native plants in alpine mining areas and to provide a scientific basis for the construction of vegetation–microbe combined restoration systems and the optimization of ecological recovery strategies in mining areas. To address these objectives, we selected seven native dominant plant species co-occurring in the Tianzhu alpine mining area and investigated their rhizosphere bacterial and fungal communities using high-throughput sequencing technology. Specifically, we analyzed the diversity, structural composition, and differentially enriched features of these communities, and predicted the ecological functions of the microbial communities. The novelty of this study lies in the systematic comparison of both bacterial and fungal communities across multiple co-occurring native plant species in the same alpine mining site—a comparative analysis that has not been previously performed in this region. This research gap is significant because understanding host-specific microbial recruitment patterns among coexisting native plants is essential for designing multi-species restoration strategies that leverage functional complementarity of rhizosphere microbiomes.

## 2. Materials and Methods

### 2.1. Sample Collection

Test samples were collected in July 2024 from Tianzhu Tibetan Autonomous County, Wuwei City, Gansu Province (36°53′41″ N, 102°41′0″ E, altitude 2616.5 m). All samples were collected from the same mining site within a 100 m × 100 m area to minimize spatial heterogeneity. The distances between individual sampling points ranged from 5 to 50 m. Based on field vegetation surveys, seven native dominant plant species with high aboveground biomass, uniform growth, and strong adaptability were selected as research subjects, namely *Leontopodium souliei* (LS), *Allium przewalskianum* (AP), *Elymus sibiricus* (ES), *Stipa przewalskyi* (SP), *Dracocephalum heterophyllum* (DH), *Silene uralensis* (SU), and *Dasiphora fruticosa* (DF). All specimens were identified by morphological observation combined with DNA barcoding technology [14,15]. Unvegetated bare degraded soil served as a baseline reference (TZ) for the unvegetated condition, rather than a strict experimental control. Bare-soil sampling points were located within 10 m of plant sampling points and collected at the same depth (0–30 cm). For each species, five healthy mature individuals were randomly selected. Root systems and rhizosphere soil samples were collected from the 0–30 cm depth. Loosely attached soil was removed by shaking, and soil firmly adhering to the root surface was collected as rhizosphere soil. The five individuals per species were pooled to form one composite sample, from which three subsamples were taken as technical replicates (treated as biological replicates for sequencing purposes, *n* = 3 per group) [16]. Composite samples were cleared of large particles and impurities. All samples were immediately stored on dry ice, rapidly transported to the laboratory, and preserved at −80 °C for subsequent sequencing.

### 2.2. DNA Extraction and PCR Amplification

Genomic DNA was extracted from all soil samples using the MagPure Soil DNA LQ Kit according to the manufacturer’s instructions. DNA concentration, purity, and integrity were assessed using a NanoDrop 2000 spectrophotometer (Thermo Fisher Scientific, Waltham, MA, USA) and agarose gel electrophoresis. Qualified DNA samples were stored at −20 °C. Using the genomic DNA as a template, PCR amplification was performed with barcode-tagged specific primers and Takara ExTaq high-fidelity enzyme. The bacterial 16S rRNA V3-V4 regions were amplified with primers 343F (5′-TACGGRAGGCAGCAG-3′) and 798R (5′-AGGGTATCTAATCCT-3′) [17]. The fungal ITS1 region was amplified using primers ITS1F (5′-CTTGGTCATTTAGAGGAAGTAA-3′) [18] and ITS2 (5′-GCTGCGTTCTTCATCGATGC-3′) [19]. After quality verification of all PCR products, high-throughput sequencing was performed on the Illumina NovaSeq 6000 platform by Shanghai Oebiotech Co., Ltd. (Shanghai, China).

### 2.3. Sequencing Data Processing and Bioinformatics Analysis

Cutadapt software (version 4.9) was used to remove primer sequences, adapters, and low-quality bases from the raw sequencing reads, completing the quality control of raw sequences. The DADA2 (version 1.28.0) algorithm was then applied to filter, denoise, merge paired-end reads, and remove chimeras from the quality-controlled sequences, yielding amplicon sequence variant (ASV) representative sequences and an abundance matrix [20]. ASVs were retained if they were present in at least two samples with a total abundance ≥ 10 reads. Species annotation of ASV representative sequences was performed by alignment against the Silva database (version 138.1, 99% similarity) using QIIME 2 software (version 2023.7) [21].

### 2.4. Statistical Analysis

Alpha diversity indices (Chao1, ACE, Shannon, Simpson) were calculated [22,23]. Normality and homogeneity of variance were tested using Shapiro–Wilk and Levene’s tests, respectively. For normally distributed data with homogeneous variances, one-way ANOVA followed by Tukey’s HSD post hoc test was applied; otherwise, Kruskal–Wallis followed by Dunn’s post hoc test was used. For two-group comparisons, Student’s *t*-test or Wilcoxon rank-sum test was applied as appropriate. Beta diversity was visualized using PCoA based on unweighted UniFrac distances. PERMANOVA (adonis2, 999 permutations) and ANOSIM (999 permutations) were used to test for significant differences and the strength of separation in community composition among groups, respectively. PERMDISP (betadisper) was additionally applied to test for heterogeneity in group dispersions. Pairwise PERMANOVA comparisons were performed with Benjamini–Hochberg FDR correction. LEfSe analysis was conducted with an LDA threshold of 4.0 and *p* < 0.05 to identify differentially enriched taxa. All multiple comparisons were corrected using the Benjamini–Hochberg FDR method, with adjusted *p* < 0.05 considered statistically significant. Functional predictions of bacterial and fungal communities were performed using the FAPROTAX and FUNGuild databases, respectively. For FUNGuild, only assignments with confidence levels of “Probable” or “Highly probable” were retained; “Possible” assignments were excluded to minimize false positives. The proportion of ASVs/sequences successfully assigned to functional categories was reported.

## 3. Results

### 3.1. Distribution Characteristics of ASVs in Root-Associated Microbial Communities of Different Plant Species

**Figure 1:**
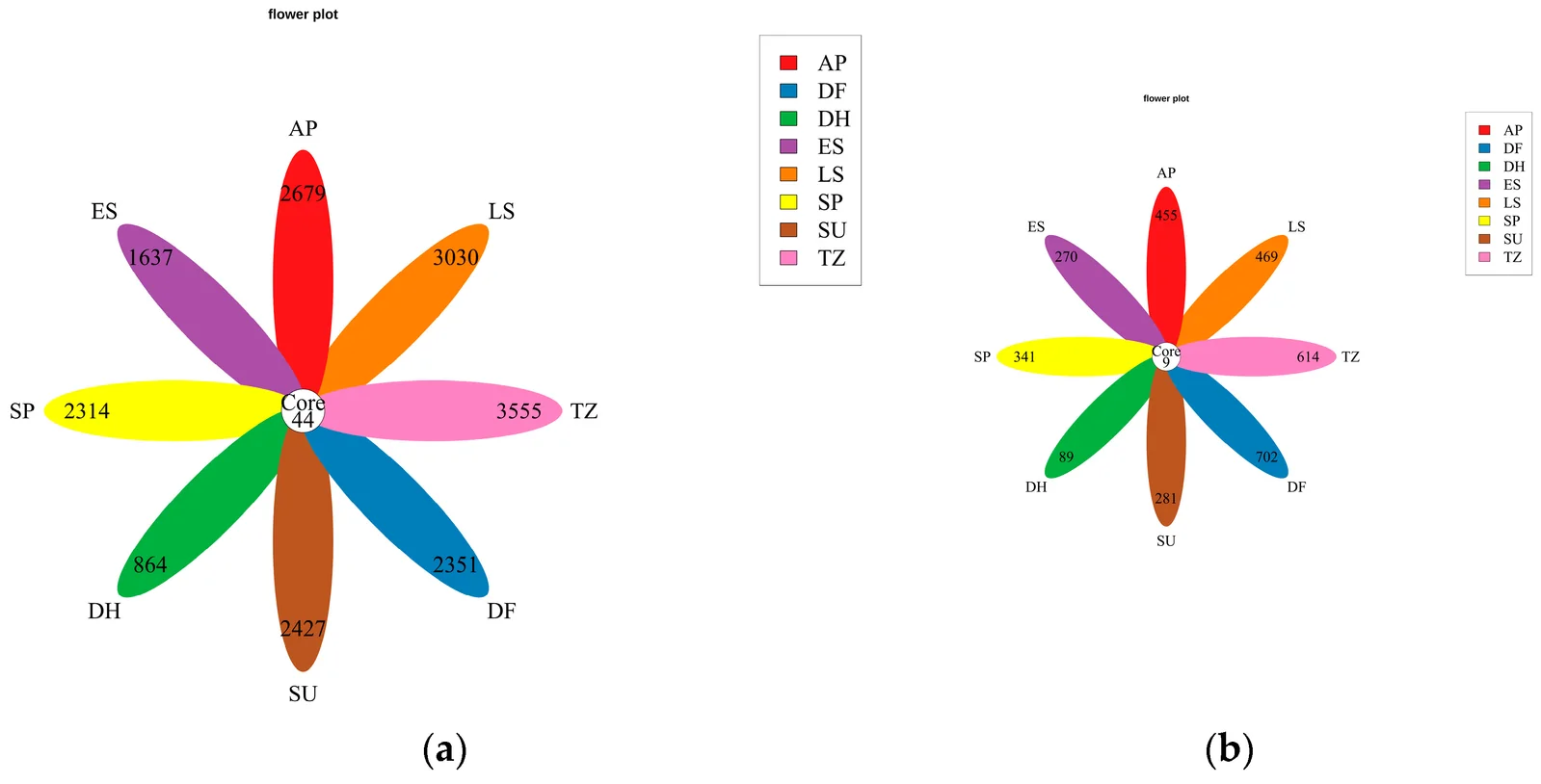
Petal plot showing ASV numbers in soil samples. Different colors represent different plant samples. LS-*Leontopodium souliei*; AP-*Allium przewalskianum*; ES-*Elymus sibiricus*; SP-*Stipa przewalskyi*; DH-*Dracocephalum heterophyllum*; SU-*Silene uralensis*; DF-*Dasiphora fruticosa*; (**a**) Bacterial ASV petal plot; (**b**) Fungal ASV petal plot.

Significant differences were observed in the number of bacterial and fungal ASVs among rhizosphere soil and bare soil (Figure 1a,b). The number of unique bacterial ASVs inTZ soil was higher than in most vegetated samples, indicating a more dispersed bacterial composition in unvegetated soil. Similarly, the number of unique fungal ASVs in TZ soil also showed distinct patterns compared to vegetated samples. The number of core microbial taxa shared across all samples was extremely low, with only 44 bacterial ASVs and 9 fungal ASVs detected as common to all samples. This indicates that plant colonization was associated with differences in community composition, and each sample exhibited strong specificity. Vegetation type was a key factor associated with the differentiation of rhizosphere microbial species composition.

### 3.2. Analysis of Biodiversity Differences in Rhizosphere Soils of Different Plant Species

#### 3.2.1. Alpha Diversity Analysis

**Figure 2:**
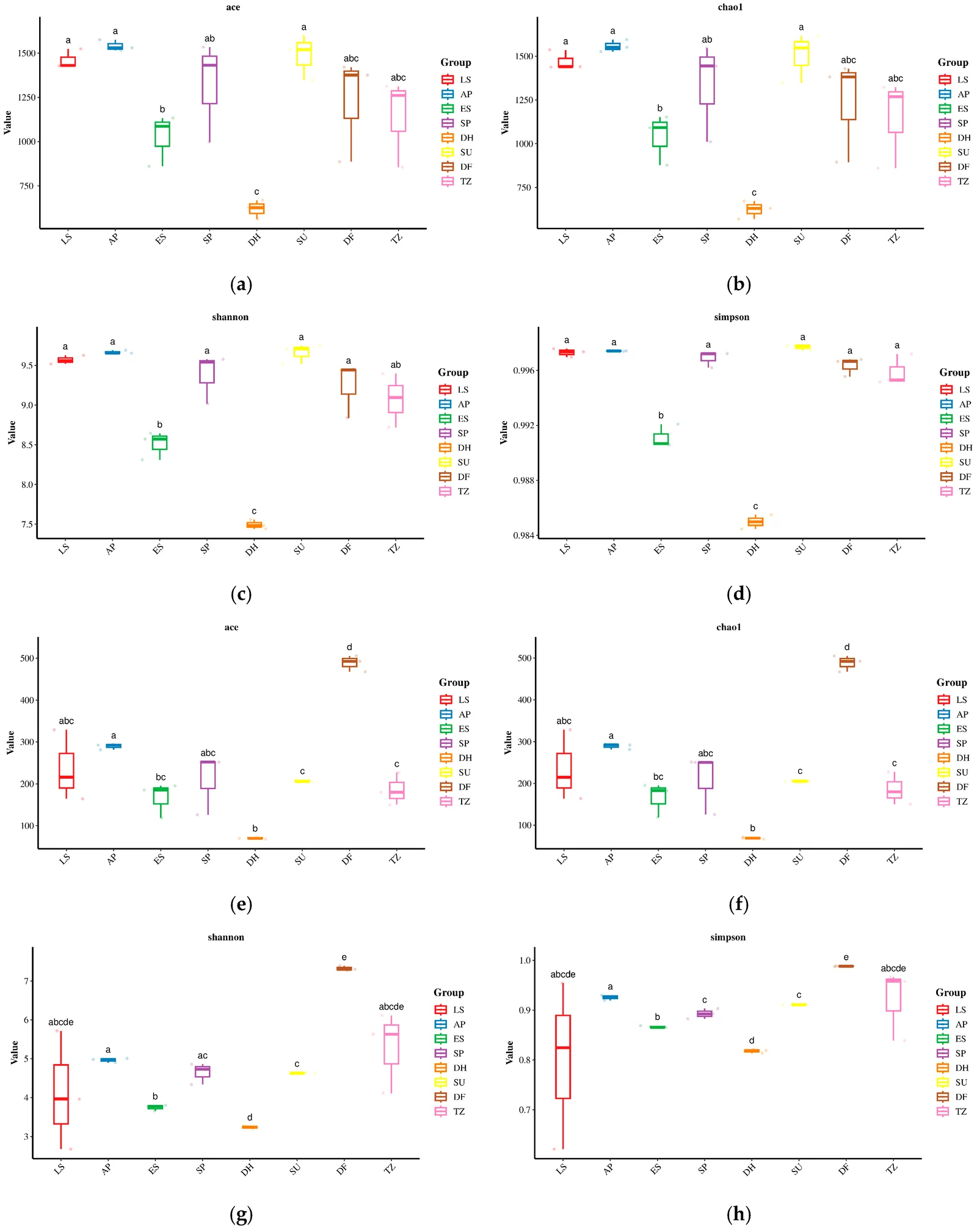
Alpha Diversity analysis of rhizosphere soil microbial communities associated with different plant species. LS-*Leontopodium souliei*; AP-*Allium przewalskianum*; ES-*Elymus sibiricus*; SP-*Stipa przewalskyi*; DH-*Dracocephalum heterophyllum*; SU-*Silene uralensis*; DF-*Dasiphora fruticosa*; Different lowercase letters above the bars indicate statistically significant differences between groups (*p* < 0.05), whereas the same lowercase letters indicate no significant difference. (**a**) ACE index of the bacterial community; (**b**) Chao1 index of the bacterial community; (**c**) Shannon index of the bacterial community; (**d**) Simpson index of the bacterial community; (**e**) ACE index of the fungal community; (**f**) Chao1 index of the fungal community; (**g**) Shannon index of the fungal community; (**h**) Simpson index of the fungal community.

All samples showed Good’s coverage > 99%, indicating adequate sequencing depth for diversity estimation. Bacterial alpha diversity varied significantly among plant species (ANOVA/Kruskal–Wallis with FDR correction, *p* < 0.05). AP rhizosphere showed the highest Chao1 and Shannon indices for bacteria (Chao1 = 1553.25 ± 35.89, Shannon = 9.67 ± 0.02). Fungal alpha diversity also showed significant variation among plant species, with DF exhibiting the highest fungal diversity (Chao1 = 488.56 ± 18.71, Shannon = 7.32 ± 0.06, Simpson = 0.99 ± 0.001, ACE = 487.82 ± 24.13) (Table 1). Cross-kingdom comparisons of alpha diversity indices were not performed due to inherent differences in primer specificity, amplification efficiency, and reference databases between 16S rRNA and ITS1 markers. The overall microbial diversity of bare soil was lower than that of most vegetated rhizosphere samples (Figure 2a–h).

#### 3.2.2. Beta Diversity Analysis of Rhizosphere Microbial Communities

**Figure 3:**
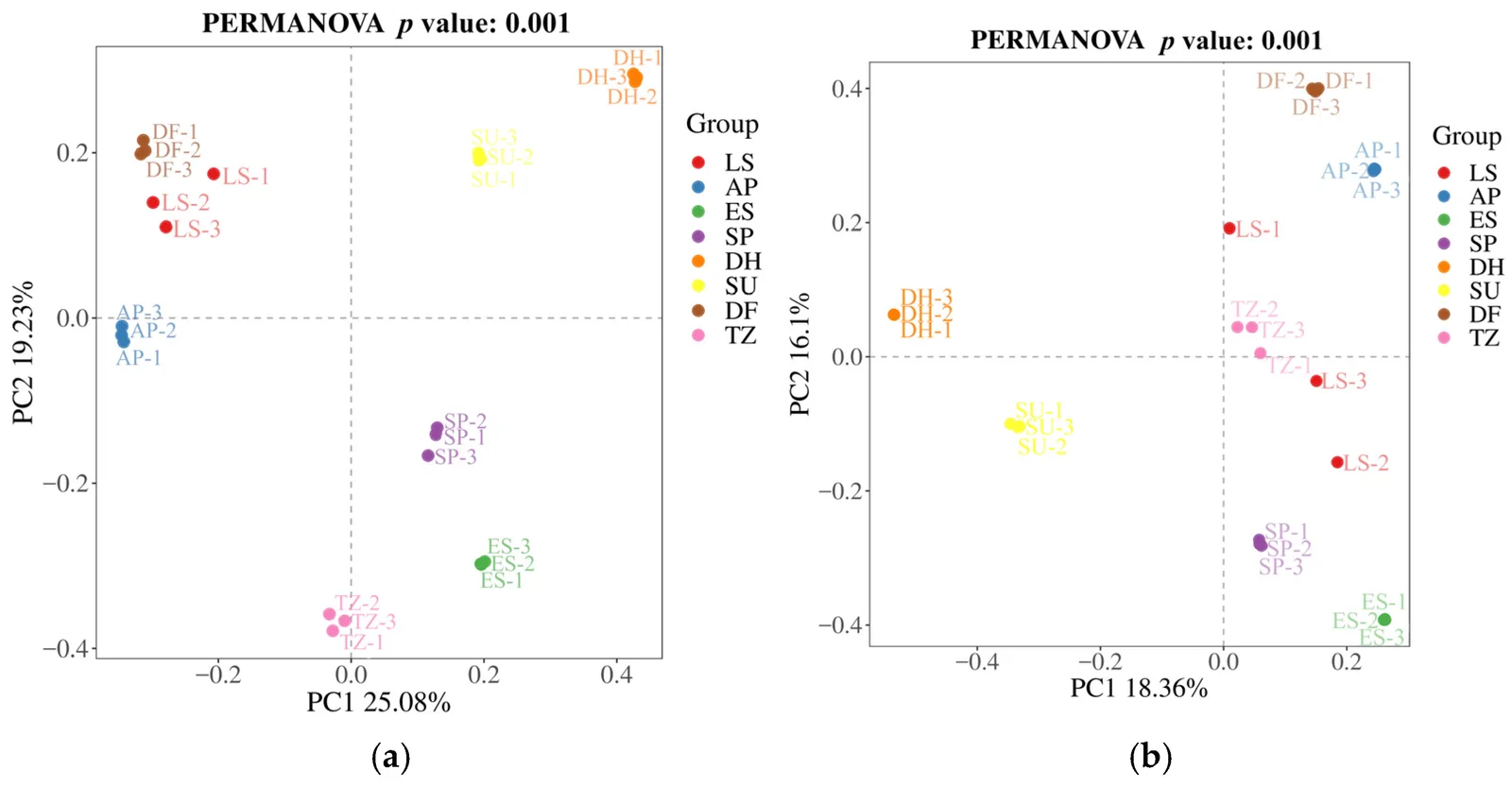
Principal coordinate analysis (PCoA) of microbial communities in rhizosphere soils. LS-*Leontopodium souliei*; AP-*Allium przewalskianum*; ES-*Elymus sibiricus*; SP-*Stipa przewalskyi*; DH-*Dracocephalum heterophyllum*; SU-*Silene uralensis*; DF-*Dasiphora fruticose*. The horizontal axis (PC1) and vertical axis (PC2) represent the two principal coordinates that explain the greatest variance among samples. Points of the same color belong to the same group, with each point representing one sample; samples with similar community composition cluster together. (**a**) Bacterial communities; (**b**) Fungal communities.

Principal coordinate analysis (PCoA) based on unweighted UniFrac distances showed that the first two axes explained 44.31% (bacteria) and 34.46% (fungi) of total variance (Figure 3a,b). Statistical tests based on Bray–Curtis dissimilarities further confirmed these patterns: ANOSIM analysis further revealed highly significant separation among plant species for both bacterial (R = 1.0, *p* < 0.001) and fungal communities (R = 0.9388, *p* < 0.001). PERMANOVA confirmed significant differences in community composition among plant species (bacteria: R^2^ = 0.78, *p* < 0.001; fungi: R^2^ = 0.84, *p* < 0.001). Additionally, PERMDISP tests showed no significant differences in multivariate dispersion among groups (bacteria: *p* > 0.05; fungi: *p* > 0.05), indicating that the PERMANOVA results primarily reflected differences in group centroids rather than within-group variability.

### 3.3. Analysis of Microbial Community Composition and Structure

**Figure 4:**
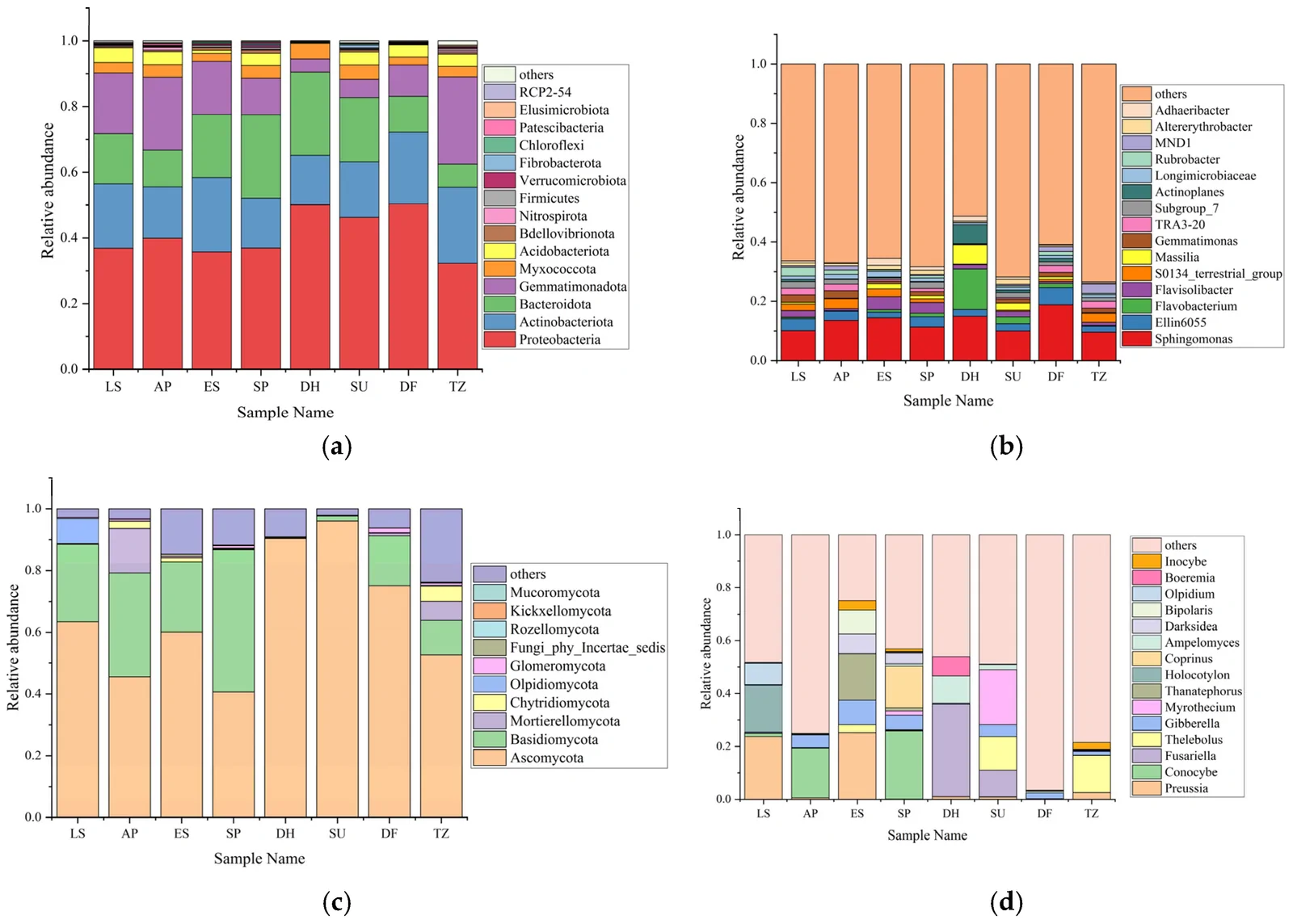
Community abundance at the phylum and genus levels. LS-*Leontopodium souliei*; AP-*Allium przewalskianum*; ES-*Elymus sibiricus*; SP-*Stipa przewalskyi*; DH-*Dracocephalum heterophyllum*; SU-*Silene uralensis*; DF-*Dasiphora fruticosa*; (**a**) Bacterial phylum; (**b**) Bacterial genus; (**c**) Fungal phylum; (**d**) Fungal genus.

Across all samples, bacterial communities comprised 33 phyla, 78 classes, 193 orders, 303 families, 531 genera, and 985 species. At the phylum level, taxa with relative abundances greater than 1% included *Proteobacteria* (32.22–50.32%), *Actinobacteriota* (15.04–23.19%), *Bacteroidota* (7.08–25.39%), *Gemmatimonadota* (3.99–26.52%), *Myxococcota* (2.38–4.79%), and *Acidobacteriota* (0.14–4.48%). *Proteobacteria* was the absolutely dominant phylum, exhibiting the highest relative abundance in DF (50.32%), followed by DH (50.10%) (Figure 4a). At the genus level, *Sphingomonas* dominated, with other abundant genera including Ellin6055, *Flavobacterium*, Flavisolibacter, S0134_terrestrial_group, *Massilia*, and *Gemmatimonas* (Figure 4b).

Fungal communities comprised 11 phyla, 34 classes, 77 orders, 171 families, 305 genera, and 355 species. At the phylum level, *Ascomycota* (45.54–96.07%) was dominant, followed by *Basidiomycota* (0.23–46.17%). *Chytridiomycota* and *Glomeromycota* were detected at lower abundances (Figure 4c). At the genus level, significant differences were observed among *Preussia*, *Thelebolus*, *Gibberella*, and *Ampelomyces* (Figure 4d). Overall, bacterial communities showed highly similar phylum-level compositions among plant species, differing only in relative abundances. In contrast, fungal communities showed more pronounced host-specific differentiation at both phylum and genus levels.

### 3.4. LEfSe Enrichment Analysis of Differential Microbial Taxa

**Figure 5:**
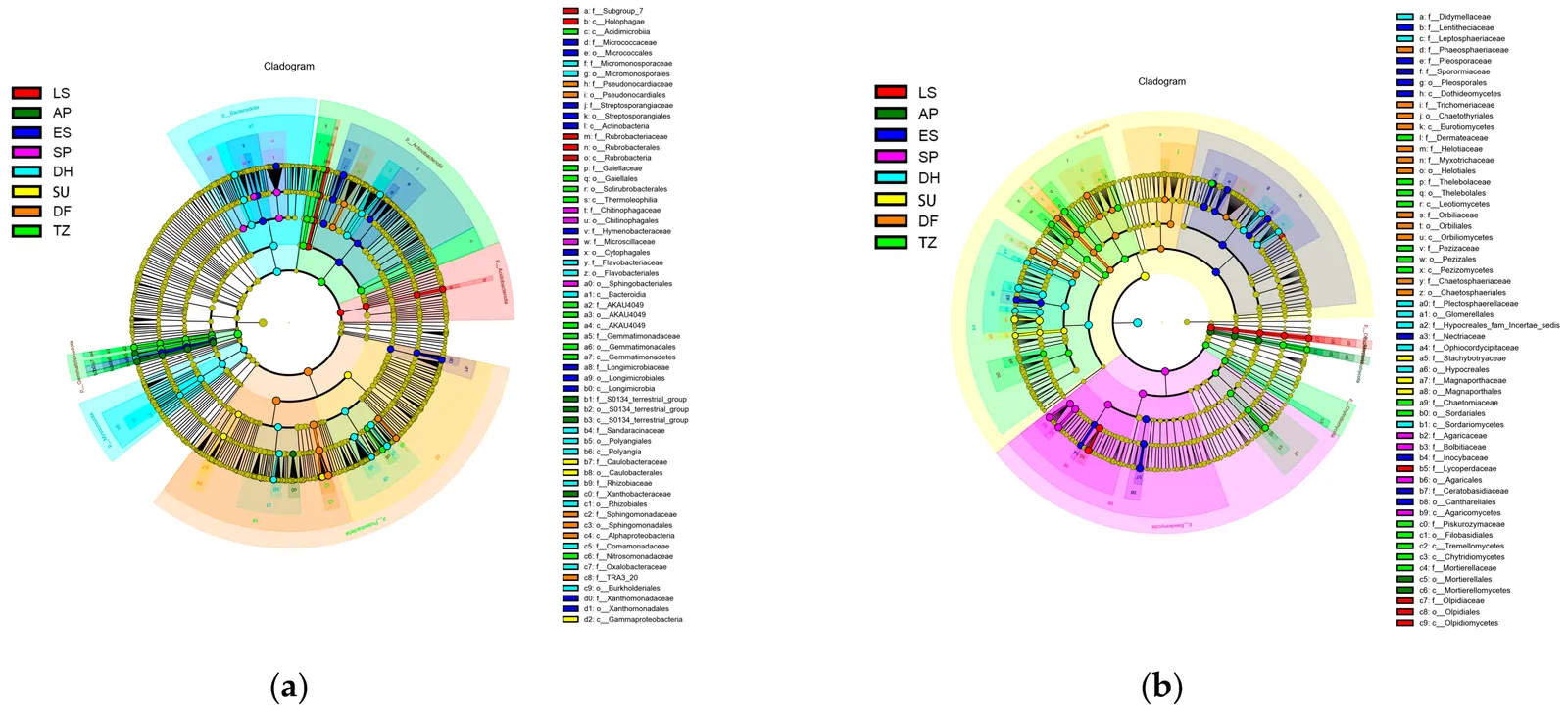
Cladogram of differentially abundant microbial taxa from LEfSe analysis. The circles radiating from the inside out in the figure represent the taxonomic levels from phylum to genus (or species). At each taxonomic level, each small circle represents a classification at that level, with the diameter of the small circle being proportional to its relative abundance. Differential species were colored according to different groupings, with LDA > 4 used as the screening criterion. The coloring principle is as follows: species with no significant differences are uniformly colored yellow, while biomarker species with significant differences are colored according to their respective groups. Nodes with different colors indicate microbial taxa that play important roles in different sample groups. The species names represented by the English letters in the figure are displayed in the legend on the right. LS-*Leontopodium souliei*; AP-*Allium przewalskianum*; ES-*Elymus sibiricus*; SP-*Stipa przewalskyi*; DH-*Dracocephalum heterophyllum*; SU-*Silene uralensis*; DF-*Dasiphora fruticosa*; (**a**) Bacterial LEfSe analysis results; (**b**) Fungal LEfSe analysis results.

LEfSe analysis revealed that different plant species were associated with distinct biomarker taxa (Figure 5a,b). LS was associated with five taxa including Subgroup_7, *Rubrobacteriaceae*, *Lycoperdaceae*, *Olpidiaceae*, and *Pseudocoleophoma*. AP was associated with four taxa including S0134_terrestrial_group, *Gemmatimonas*, *Xanthobacteraceae*, and *Mortierellales*. ES was associated with 12 taxa including *Streptosporangiaceae*, *Longimicrobiaceae*, *Xanthomonadaceae*, *Micrococcaceae*, *Flavisolibacter*, *Hymenobacteraceae*, *Sporormiaceae*, *Ceratobasidiaceae*, *Nectriaceae*, *Pleosporaceae*, *Lentitheciaceae*, and *Inocybaceae*. SP was associated with five taxa including *Chitinophagaceae*, *Microscillaceae*, *Sphingobacteriales*, *Bolbitiaceae*, and *Agaricaceae*. A total of 14 taxa were enriched in BHHZZ: *Flavobacteriaceae*, *Micromonosporaceae*, *Oxalobacteraceae*, *Rhizobacter*, *Rhizobiaceae*, *Comamonadaceae*, *Allorhizobium-Neorhizobium-Pararhizobium-Rhizobium*, *Sandaracinaceae*, *Longimicrobium*, *Didymellaceae*, *Leptosphaeriaceae*, *Plectosphaerellaceae*, *Ophiocordycipitaceae* and *Hypocreales*_fam_Incertae_sedis. SU was associated with six taxa including *Novosphingobium*, *Caulobacteraceae*, *Gammaproteobacteria*, *Devosia*, *Stachybotryaceae*, and *Magnaporthaceae*. DF was associated with ten taxa including *Sphingomonadaceae*, *Pseudonocardiaceae*, Ellin6055, TRA3_20, *Phaeosphaeriaceae*, *Trichomeriaceae*, *Helotiaceae*, *Myxotrichaceae*, *Orbiliaceae*, and *Chaetosphaeriaceae*. TZ was characterized by 12 taxa including *Gemmatimonadaceae*, *Acidimicrobiia*, *Gaiellaceae*, *Nitrosomonadaceae*, *Solirubrobacterales*, AKAU4049, *Thelebolaceae*, *Mortierellaceae*, *Dermateaceae*, *Pezizaceae*, *Chaetomiaceae*, and *Piskurozymaceae*. The differential taxa were mainly affiliated with core phyla including *Acidobacteriota*, *Actinobacteriota*, *Gemmatimonadota*, *Proteobacteria*, *Bacteroidota*, *Myxococcota*, *Basidiomycota*, *Olpidiomycota*, *Ascomycota*, and *Mortierellomycota*. These taxa represent candidate functional groups that may merit further investigation for their roles in soil amelioration and stress resistance, but their actual functions in situ require experimental validation.

### 3.5. Functional Prediction Analysis of Rhizosphere Microorganisms

#### 3.5.1. Bacterial Functional Prediction Based on the FAPROTAX Database

**Figure 6:**
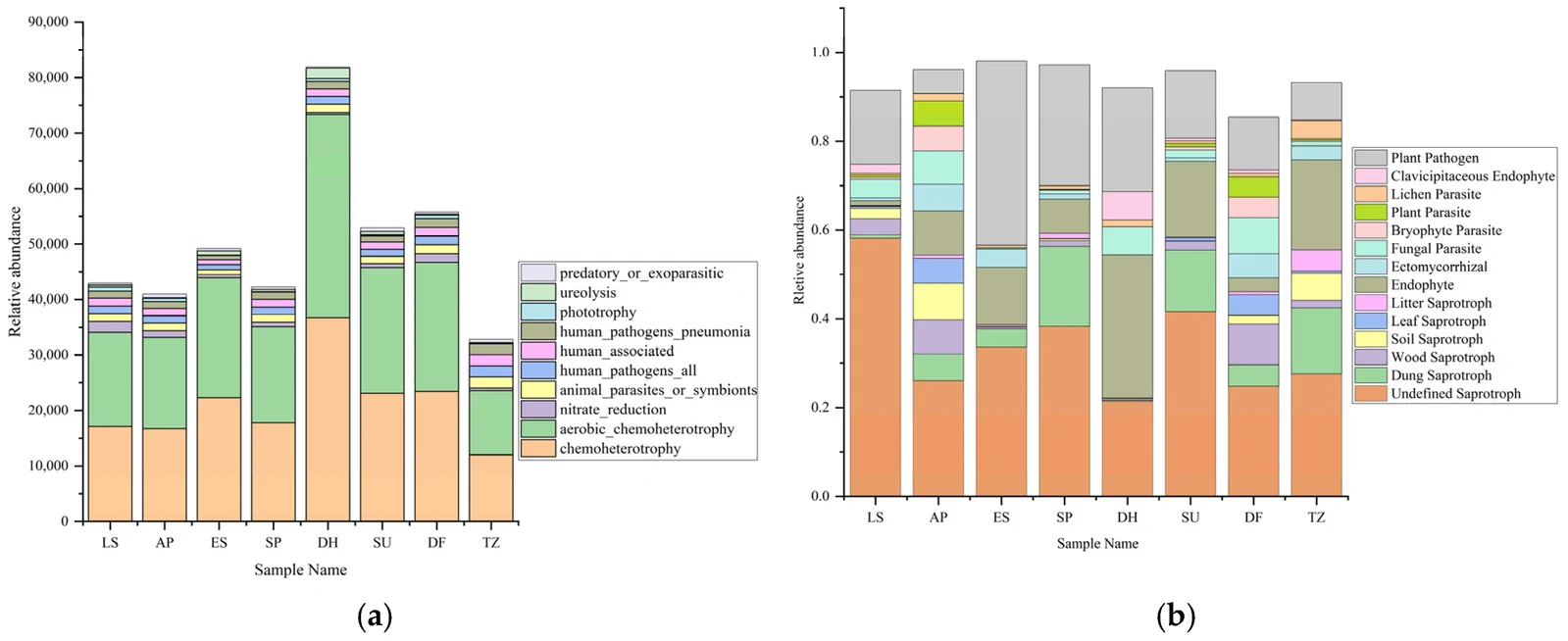
Functional abundance of microbial communities in rhizosphere soils of different plant species. LS-*Leontopodium souliei*; AP-*Allium przewalskianum*; ES-*Elymus sibiricus*; SP-*Stipa przewalskyi*; DH-*Dracocephalum heterophyllum*; SU-*Silene uralensis*; DF-*Dasiphora fruticosa*; (**a**) Bacterial functional prediction based on FAPROTAX; (**b**) Fungal functional prediction based on FUNGuild.

The core bacterial functions were chemoheterotrophy and aerobic chemoheterotrophy, together accounting for >50% of relative abundance (Figure 6a). The relative abundances of chemoheterotrophy- and aerobic chemoheterotrophy-associated categories varied among plant species, with DH rhizosphere showing the highest relative abundances, suggesting a potential association with organic matter turnover that warrants experimental validation. Functions such as nitrate reduction and ureolysis were also detected at variable abundances across species. The relative abundances of animal parasitism- and pathogen-related functional categories were lower in vegetated soils than in bare soil, but these assignments are predictive and should not be interpreted as direct evidence of in situ suppression.

#### 3.5.2. Fungal Functional Prediction Based on the FUNGuild Database

Fungal function communities were classified into three trophic modes: saprotrophs, symbiotrophs, and pathotrophs, with significant host-specific variation (Figure 6b). Saprotrophs constituted the dominant trophic mode across all samples, with the highest relative abundance observed in LS and the lowest in DH. Symbiotrophs were relatively more abundant in certain species; notably, AP displayed the highest mycorrhizal abundance, whereas DH was enriched in endophytic fungi. Pathotroph-related guilds consistently accounted for less than 5% of total assignments across all samples. Nevertheless, these functional assignments are predictive in nature and should be interpreted with caution, as they do not constitute direct evidence of disease risk.

## 4. Discussion

### 4.1. Ecological Value of Native Plant-Microbe Synergistic Remediation in Alpine Mining Areas

Alpine mining areas are subjected to multiple stresses—high-altitude low temperatures, soil impoverishment, structural degradation, and long-term anthropogenic disturbance—resulting in extremely poor ecosystem stability. The exhaustion of soil microecological functions and difficulties in vegetation colonization constitute core bottlenecks for regional restoration [24,25]. Compared with artificial soil improvement or exogenous fertilization, reshaping rhizosphere microecology through native plants and harnessing plant–microbe synergies for natural soil restoration represents a low-cost, sustainable, and ecologically authentic remediation model [26,27]. The diversity, structure, and function of rhizosphere microbial communities directly determine the efficiency of material cycling and vegetation recovery, serving as core drivers of positive ecological succession [28]. This study systematically characterized community traits and functional differentiation of rhizosphere microorganisms of seven native species, described host plant-associated patterns, and identified candidate core functional groups with remediation potential, providing baseline information for vegetation–microbe combined strategies in the Qilian Mountains alpine mining areas.

### 4.2. Host-Associated Differences in Rhizosphere Microbial Diversity

Rhizosphere microbial diversity showed significant host specificity: AP had the highest bacterial diversity, DF showed prominent fungal dominance, while DH had the lowest overall diversity. Bare soil microecological structure and functions were significantly inferior to vegetated rhizosphere, suggesting that vegetation colonization is important for activating soil microbial activity and enhancing diversity. In nutrient-poor habitats, different plant species selectively influence functional microbial communities through differential regulation of root exudates, rhizosphere pH, and redox potential, establishing customized rhizosphere adaptation systems [29,30]. Beta diversity (PCoA) further validated that LS, AP, and DF had relatively similar community structures, while DH and SU displayed notable specificity. These findings indicate that the seven species were associated with differentiated microbial recruitment patterns, with potential functional complementarity among species-specific microbiota, suggesting that multi-species mixed planting may achieve comprehensive functional coverage [31].

### 4.3. Core Communities and Candidate Biomarker Taxa

Species composition analysis showed that *Proteobacteria*, *Actinobacteriota*, and *Bacteroidota* were core bacterial groups, and *Ascomycota* dominated fungi. These are considered stress-tolerant and ecologically stable indigenous taxa in alpine degraded soils, demonstrating high adaptability to the extreme conditions of alpine mining areas [32]. The specific biomarker taxa identified by LEfSe showed distinct enrichment patterns. For instance, *Flavobacteriaceae* and *Oxalobacteraceae* were enriched in the DH rhizosphere. Previous studies have reported that members of these families possess organic matter decomposition and soil conditioning functions [33]; however, their actual activities in the present study remain to be experimentally confirmed. Similarly, *Gemmatimonas* was enriched in the AP rhizosphere, a genus previously reported to promote nitrogen retention and soil fertility [34], but direct experimental validation from this study is required. *Sphingomonadaceae* enriched in DF rhizosphere have been reported to include stress-tolerant and growth-promoting members [35,36], but these functions were not directly tested here. The absence of marked enrichment of efficient functional groups in bare soil appears consistent with the view that vegetation-mediated rhizosphere processes might influence soil microecology. Importantly, the taxonomic enrichments observed in this study should be interpreted as statistical associations rather than functional demonstrations.

### 4.4. Functional Predictions and Candidate Functional Complementarity

The FAPROTAX-predicted functional profiles suggested that bacterial communities were predominantly heterotrophic. The higher relative abundance of chemoheterotrophy-associated categories in DH rhizosphere may indicate a potential for organic matter turnover, but this interpretation is based on prediction and requires experimental validation. LS showed higher relative abundance of nitrate reduction-associated categories, which may suggest a potential for alleviating nitrogen limitation [37]. Fungal communities exhibited a trophic structure dominated by saprotrophs, supplemented by symbiotrophs, with a very low proportion of pathotrophs [38]. Mycorrhizal fungi associated with AP rhizosphere may enhance nutrient and water uptake, while endophytic fungi in DH rhizosphere may improve tolerance to heavy metals and low temperature [39]. The rhizosphere microbiota collectively formed a comprehensive functional system potentially covering soil amelioration, nutrient mobilization, stress resistance, growth promotion, and microecological purification. However, it is critical to emphasize that all functional interpretations in this study are based on predictive algorithms (FAPROTAX and FUNGuild) rather than direct measurements of microbial activity. The presence of taxa associated with certain functional guilds in the previous literature does not demonstrate that these functions are being actively performed in situ. Therefore, our functional predictions should be viewed as hypothesis-generating rather than evidence of actual ecological processes.

### 4.5. Study Limitations

Several limitations should be considered when interpreting our findings. First, the cross-sectional single-time-point design captures community profiles at one time only and cannot assess temporal dynamics or stability. Second, bare soil and rhizosphere soil are inherently different environments; therefore, the bare-soil samples serve as a baseline reference for the unvegetated condition rather than a strict control, and vegetation effects cannot be fully disentangled from soil environmental heterogeneity. Third, the absence of soil physicochemical and heavy metal measurements limits our ability to identify the environmental drivers of microbial community assembly. Fourth, FAPROTAX and FUNGuild annotations provide predictive functional inferences based on sequence homology and database records, not direct evidence of in situ activity; taxonomic enrichment does not demonstrate functional execution. Fifth, amplicon sequencing of 16S rRNA and ITS1 markers has inherent constraints in taxonomic resolution and functional inference. These limitations indicate that our results should be viewed as hypothesis-generating rather than conclusive. Future studies incorporating temporal sampling, comprehensive soil physicochemical and heavy metal analyses, metagenomics, and strain isolation/inoculation experiments are needed to validate the candidate taxa and plant–microbe associations identified here [40,41].

## 5. Conclusions

In summary, this study systematically characterized the diversity, composition, and predicted functional profiles of rhizosphere bacterial and fungal communities associated with seven native plant species in the Tianzhu alpine mining area. The result demonstrate that rhizosphere microbial community composition differs significantly among plant species, with *Proteobacteria*, *Actinobacteriota*, and *Ascomycota* as the core phyla. Certain taxa (e.g., *Flavobacteriaceae*, *Gemmatimonas*, *Sphingomonadaceae*) were differentially enriched in specific plant rhizospheres and showed statistical associations with predicted functional categories related to nutrient cycling, stress tolerance, and organic matter turnover. These findings provide baseline data and candidate targets for future mechanistic studies and restoration-oriented investigations. However, because our study is observational and cross-sectional, and functional predictions remain to be experimentally validated, we emphasize that the observed patterns are correlative rather than causal. The proposed vegetation–microbe combined restoration concept should be viewed as a hypothesis inspired by our data, not a demonstrated application. Future experimental validation through strain isolation, physiological assays, and inoculation experiments is essential before any restoration applications can be considered.

## Figures and Tables

**Table 1:** Alpha diversity indices of bacteria and fungi in rhizosphere soils of different plants.

Microorganism	Sample Name	Chao1	Shannon	Simpson	ACE
Bacteria	LS	1472.58 ^ab^ ± 26.69	9.56 ^a^ ± 0.05	0.9976 ^a^ ± 0.00	1464.65 ^a^ ± 63.65
	AP	1553.25 ^a^ ± 35.89	9.67 ^a^ ± 0.02	0.9973 ^a^ ± 0.00	1539.97 ^a^ ± 29.20
	ES	1038.66 ^c^ ± 143.13	8.51 ^c^ ± 0.18	0.9913 ^c^ ± 0.00	972.57 ^c^ ± 153.65
	SP	1329.82 ^abc^ ± 283.36	9.38 ^ab^ ± 0.31	0.9969 ^ab^ ± 0.00	1479.99 ^abc^ ± 68.27
	DH	623.39 ^d^ ± 50.44	7.49 ^d^ ± 0.06	0.9850 ^d^ ± 0.00	616.17 ^d^ ± 46.94
	SU	1502.65 ^ab^ ± 139.85	9.65 ^a^ ± 0.12	0.9978 ^a^ ± 0.00	1562.49 ^ab^ ± 55.48
	DF	1235.80 ^abcd^ ± 296.25	9.24 ^ab^ ± 0.35	0.9963 ^ab^ ± 0.00	1225.22 ^abcd^ ± 294.82
	TZ	1223.80 ^bc^ ± 209.60	9.14 ^b^ ± 0.28	0.9953 ^b^ ± 0.00	1213.33 ^bc^ ± 204.21
Fungal	LS	236.87 ^bcd^ ± 86.14	4.12 ^abcde^ ± 1.52	0.72 ^abcde^ ± 0.17	234.78 ^bcd^ ± 88.19
	AP	289.11 ^b^ ± 7.98	4.96 ^b^ ± 0.06	0.93 ^b^ ± 0.005	291.09 ^b^ ± 5.06
	ES	165.92 ^cd^ ± 41.05	3.75 ^d^ ± 0.08	0.86 ^d^ ± 0.002	168.47 ^cd^ ± 45.30
	SP	210.80 ^bcd^ ± 73.53	4.64 ^bc^ ± 0.27	0.89 ^c^ ± 0.01	189.86 ^bcd^ ± 89.36
	DH	69.66 ^d^ ± 2.22	3.24 ^e^ ± 0.04	0.82 ^e^ ± 0.003	72.47 ^d^ ± 0.87
	SU	206.80 ^c^ ± 2.79	4.63 ^c^ ± 0.02	0.91 ^c^ ± 0.001	207.64 ^c^ ± 5.50
	DF	488.56 ^a^ ± 18.71	7.32 ^a^ ± 0.06	0.99 ^a^ ± 0.001	487.82 ^a^ ± 24.13
	TZ	175.74 ^c^ ± 33.58	4.59 ^bcde^ ± 1.29	0.89 ^abcde^ ± 0.14	175.62 ^c^ ± 32.82

LS-*Leontopodium souliei*; AP-*Allium przewalskianum*; ES-*Elymus sibiricus*; SP-*Stipa przewalskyi*; DH-*Dracocephalum heterophyllum*; SU-*Silene uralensis*; DF-*Dasiphora fruticosa*; Mean ± standard deviation, *n* = 3; Different lowercase letters indicate significant differences at *p* < 0.05 level between treatments.

## Data Availability

The original contributions presented in this study are included in the article. Further inquiries can be directed to the corresponding authors.

## Abbreviations

The following abbreviations are used in this manuscript:

LS	*Leontopodium souliei*
AP	*Allium przewalskianum*
ES	*Elymus sibiricus*
SP	*Stipa przewalskyi*
DH	*Dracocephalum heterophyllum*
SU	*Silene uralensis*
DF	*Dasiphora fruticosa*
TZ	Bare soil baseline reference
